# Molecular insights into the responses of barley to yellow mosaic disease through transcriptome analysis

**DOI:** 10.1186/s12870-023-04276-x

**Published:** 2023-05-19

**Authors:** Mengna Zhang, Yi Hong, Juan Zhu, Yuhan Pan, Hui Zhou, Chao Lv, Baojian Guo, Feifei Wang, Rugen Xu

**Affiliations:** Key Laboratory of Plant Functional Genomics of the Ministry of Education / Jiangsu Key Laboratory of Crop Genomics and Molecular Breeding/ Jiangsu Co-Innovation Center for Modern Production Technology of Grain Crops/ Joint International Research Laboratory of Agriculture and Agri-Product Safety of Ministry of Education of China, Yangzhou University, Yangzhou, 225009 China

**Keywords:** Barley, Barley yellow mosaic virus disease, RNA-Seq, Differentially expressed genes, Natural infection

## Abstract

**Background:**

Barley (*Hordeum vulgare* L.) represents the fourth most essential cereal crop in the world, vulnerable to barley yellow mosaic virus (BaYMV) and/or barley mild mosaic virus (BaMMV), leading to the significant yield reduction. To gain a better understanding of the mechanisms regarding barley crop tolerance to virus infection, we employed a transcriptome sequencing approach and investigated global gene expression among three barley varieties under both infected and control conditions.

**Results:**

High-throughput sequencing outputs revealed massive genetic responses, reflected by the barley transcriptome after BaYMV and/or BaMMV infection. Significant enrichments in peptidase complex and protein processing in endoplasmic reticulum were clustered through Gene ontology and KEGG analysis. Many genes were identified as transcription factors, antioxidants, disease resistance genes and plant hormones and differentially expressed between infected and uninfected barley varieties. Importantly, general response genes, variety-specific and infection-specific genes were also discovered. Our results provide useful information for future barley breeding to resist BaYMV and BaMMV.

**Conclusions:**

Our study elucidates transcriptomic adaptations in barley response to BaYMV/BaMMV infection through high-throughput sequencing technique. The analysis outcome from GO and KEGG pathways suggests that BaYMV disease induced regulations in multiple molecular-biology processes and signalling pathways. Moreover, critical DEGs involved in defence and stress tolerance mechanisms were displayed. Further functional investigations focusing on these DEGs contributes to understanding the molecular mechanisms of plant response to BaYMV disease infection, thereby offering precious genetic resources for breeding barley varieties resistant to BaYMV disease.

**Supplementary Information:**

The online version contains supplementary material available at 10.1186/s12870-023-04276-x.

## Backgrounds

Barley yellow mosaic virus (BaYMV) and barley mild mosaic virus (BaMMV) belong to the genus Bymovirus in the family Potyviridae, causing yellow mosaic disease in barley and thereby resulting in severe yield losses in the susceptible cultivars [1]. Both viruses are transmitted by soil-borne plasmodiophorid *Polymyxa graminis* (*P. graminis*), and are a prevalent disease in Europe and East Asia winter barley regions [2]. Barley crops infected by BaYMV and/or BaMMV shows yellow chlorotic spots and discoloration on leaves during the vegetative growth stage [3], and till to the jointing stage, barley growth was hindered and delayed [4]. To rescue barley yield loss under these diseases, there is a need to carry out BaYMV disease research, aiming to disclose the mechanisms.

Both BaYMV and BaMMV have a single-stranded positive-sense RNA genome composed of two RNA segments, RNA1 and RNA2 [5]. While RNA1 encodes a large polyprotein, which is cleaved into 9 mature peptides (P3N-PIPO, 6K1, CI, 6K2, VPg, NIa, Pro, NIb, CP) by proteolysis, RNA2 encodes a polyprotein which is only composed by two mature peptides, named as P1 and P2 proteins [6]. To protect plants against pathogen invasion, multiple resistant strategies triggering immunities have been developed and evolved, such as pathogen-associated molecular pattern (PAMP)-triggered immunity (PTI) and effector-triggered immunity (ETI) [7, 8]. Previous studies have disclosed agricultural practices for reducing the incidence of BaYMV, including late sowing and field rotations, chemical fumigation and fungicides application. However, these methods are not very suitable (less economic and less efficient) given to the high cost in field maintenance and environmental pollution. Therefore, improving host plant resistance to BaYMV disease sits as the most economical, effective and environmentally friendly way to alleviate the negative effects of BaYMV disease on crop yield. So far, twenty-two genes associated with BaYMV disease resistance have been identified [3]. Of them, two genes (*eIF4E* and *PDIL5-1*) have been cloned and functionally characterized, located on chromosome 3HL and chromosome 4HL, respectively [4, 9]. It appeared that each chromosome (1H, 2H, 3H, 4H, 5H, 6H or 7H) contains one or more resistance genes [3]. Whether these resistance genes induce a similar or different mechanisms underlying BaYMV resistance requires experimental confirmation.

With the rapid development of RNA sequencing technology (RNA-Seq) but low costs, sequencing all transcripts (termed transcriptome, including mRNAs and non-coding RNAs) in barley become more accessible and preferred due to the its enhanced sensitivity, coverage, throughput and resolution. In recent years, transcriptome sequencing is frequently utilised to analyze the transcriptome profiles of barley under both abiotic stresses, such as drought, salinity, low nitrogen, heavy metal; and biotic stresses including powdery mildew, ramularia leaf spot disease, *Puccinia graminis f. sp. tritici* and *Ramularia collo-cygni* [10–17]. To date, this approach has become well-acknowledged and commonly applied in conducting mechanism investigations into biological and abiotic stress tolerance of crops [18, 19].

To gain a better understanding of the mechanism of barley crop against virus infection, transcriptome profiles of three selected barley varieties infected with BaYMV and BaMMV was conducted. Significant differential expressions of genes between the infected and uninfected barley cultivars, between BaYMV-infected and BaMMV-infected barley plants were demonstrated. Our results provide molecular insights into barley plant responses to infections of BaYMV and BaMMV.

## Results

### Confirmation of BaYMV disease infection in barley plants

**Fig. 1:**
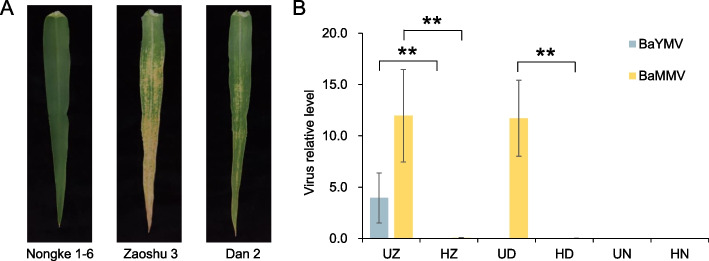
The phenotype of barley sampled. **A** Leaves phenotypes of three barley varieties after infection with BaYMV disease. **B** Leaves phenotypes of three barley varieties after infection with BaYMV disease. The blue column represents the virus relative level of BaYMV and the yellow column represents the relative viral expression of BaMMV

In the diseased field, newly emerged leaves of barley plants were found to have chlorosis and mottling symptoms and late on, the plants developed into leaf etiolation and even death, representing the typical BaYMV disease symptoms. In contrast, same cultivars grown in the adjacent healthy field indicated no symptoms (Fig. 1A). The barley cultivars Zaoshu 3 (UZ), Dan 2 (UD) and Nongke 1–6 (UN) were sampled from the diseased nursery. Meanwhile, virus-free healthy plants of the same cultivar Zaoshu 3 (HZ), Dan 2 (HD) and Nongke 1–6 (HN) were obtained from a disease-free field (less than 100 m distance) as a control. To confirm whether the plants were infected or not infected with BaYMV and/or BaMMV, qPCR analysis was performed. Primers were designed according to the coat protein sequences of BaYMV and the viral coding sequences of BaMMV, while *GADPH* and *α-Tubulin* was used as a reference gene. Three biological replicates were designed for each sample. The result from the disease field showed that BaYMV and BaMMV co-infected all plants of Zaoshu 3 (UZ) cultivar, BaMMV infected all plants of Dan 2 (UD) cultivar but none of these two viruses infected plants of Nongke 1–6 (UN) cultivar (Fig. 1B). Further comparison showed that there was a significant difference in BaYMV and BaMMV levels among the infected cultivars (Fig. 1B). While the level of BaMMV was high in both UZ and UD, the level of BaYMV was only high in UZ. In UD the level of BaYMV was almost undetectable. In addition, the level of BaMMV was much higher than that of BaYMV in UZ (Fig. 1B).

### Genome-wide sequencing of mRNAs

With the sequencing technology, each sample generated more than 42 million raw reads (Table 1). After quality control, a minimum of 41,640,002 clean reads (UZ3) were obtained for each sample. The UD3 sample generated the highest number of clean reads (55,845,286) (Table 1). GC contents of these samples ranged between 53.82% and57.84% with a mean GC contents of 55.78% (Table 1). Table 1 also shows sequencing error rates of each sample, demonstrating that all the samples had a good sequencing quality.

**Table 1:** Summary of RNA sequencing results

Sample	Raw reads	Clean reads	Clean bases (GB)	Q20 (%)	Q30 (%)	GC (%)
UZ1	46,603,148	45,146,854	6.77	98.00	94.22	56.33
UZ2	49,420,806	47,807,722	7.17	97.81	93.86	54.78
UZ3	42,902,642	41,640,002	6.25	97.53	92.90	53.82
HZ1	48,212,238	46,410,308	6.96	97.74	93.65	56.33
HZ2	48,498,062	46,806,002	7.02	97.78	93.83	56.54
HZ3	48,221,644	46,489,082	6.97	97.97	94.27	56.16
UD1	50,124,628	48,356,138	7.25	97.97	94.12	54.41
UD2	47,484,336	45,806,326	6.87	97.78	93.80	54.68
UD3	58,102,318	55,845,286	8.38	97.84	93.96	55.31
HD1	49,217,846	48,033,504	7.21	98.17	94.37	57.84
HD2	54,232,698	52,466,972	7.87	97.77	93.79	55.85
HD3	54,567,778	52,631,730	7.89	97.96	94.18	55.60
UN1	48,761,438	46,973,514	7.05	97.84	94.00	56.53
UN2	50,405,442	48,520,770	7.28	97.93	94.19	57.07
UN3	49,327,488	47,639,518	7.15	97.82	93.87	56.43
HN1	47,287,982	45,726,868	6.86	98.08	94.41	55.06
HN2	47,632,352	46,103,870	6.92	97.93	94.08	54.54
HN3	49,838,866	47,919,512	7.19	97.72	93.65	56.67

Q20 and Q30 demonstrate the sequencing error rate < 1% and < 0.1%, respectively

### Barley genome mapping of mRNA contigs

All of read contigs were mapped to the barley reference genome using HISAT software. It showed that more than 86% of clean reads could map to the barley genome and more than 76% of the reads could uniquely map to the barley genome (Table 2). Percentages of reads that mapped to the barley reference genome either in sense orientation or anti-sense orientation were similar to each other between samples (Table 2). Analysis of read distribution on the barley genome showed that exonic regions-derived reads were significantly higher than intergenic or intronic regions-derived reads (Table S1), indicating that the majority of reads came from mature mRNAs.

**Table 2:** Summary of reads aligned to the barley reference genome

Sample	Mapped reads	Unique_map	Multi_map	Positive_map	Negative_map
UZ1	42,204,013(93.48%)	36,458,616(80.76%)	5,745,397(12.73%)	18,234,761(40.39%)	18,223,855(40.37%)
UZ2	41,401,082(86.6%)	36,452,969(76.25%)	4,948,113(10.35%)	18,223,689(38.12%)	18,229,280(38.13%)
UZ3	36,043,243(86.56%)	32,037,641(76.94%)	4,005,602(9.62%)	16,014,615(38.46%)	16,023,026(38.48%)
UD1	42,259,977(87.39%)	37,232,868(77.00%)	5,027,109(10.40%)	18,620,507(38.51%)	18,612,361(38.49%)
UD2	41,110,107(89.75%)	36,684,885(80.09%)	4,425,222(9.66%)	18,342,565(40.04%)	18,342,320(40.04%)
UD3	48,810,683(87.40%)	43,433,424(77.77%)	5,377,259(9.63%)	21,727,312(38.91%)	21,706,112(38.87%)
UN1	43,486,490(92.58%)	38,030,522(80.96%)	5,455,968(11.61%)	19,032,008(40.52%)	18,998,514(40.45%)
UN2	44,843,645(92.42%)	38,502,837(79.35%)	6,340,808(13.07%)	19,270,958(39.72%)	19,231,879(39.64%)
UN3	44,030,398(92.42%)	38,281,229(80.36%)	5,749,169(12.07%)	19,159,001(40.22%)	19,122,228(40.14%)
HZ1	43,137,950(92.95%)	37,324,139(80.42%)	5,813,811(12.53%)	18,676,092(40.24%)	18,648,047(40.18%)
HZ2	43,738,955(93.45%)	37,521,433(80.16%)	6,217,522(13.28%)	18,775,838(40.11%)	18,745,595(40.05%)
HZ3	43,288,978(93.12%)	37,399,491(80.45%)	5,889,487(12.67%)	18,707,226(40.24%)	18,692,265(40.21%)
HD1	45,041,942(93.77%)	38,659,674(80.48%)	6,382,268(13.29%)	19,364,416(40.31%)	19,295,258(40.17%)
HD2	48,647,883(92.72%)	42,259,657(80.55%)	6,388,226(12.18%)	21,143,526(40.3%)	21,116,131(40.25%)
HD3	49,234,196(93.54%)	42,446,974(80.65%)	6,787,222(12.9%)	21,234,408(40.35%)	21,212,566(40.30%)
HN1	42,887,557(93.79%)	36,512,963(79.85%)	6,374,594(13.94%)	18,274,284(39.96%)	18,238,679(39.89%)
HN2	43,101,345(93.49%)	37,514,180(81.37%)	5,587,165(12.12%)	18,765,726(40.70%)	18,748,454(40.67%)
HN3	44,524,423(92.92%)	38,235,277(79.79%)	6,289,146(13.12%)	19,140,629(39.94%)	19,094,648(39.85%)

### Differential expression of genes in barley grown under different conditions

**Fig. 2:**
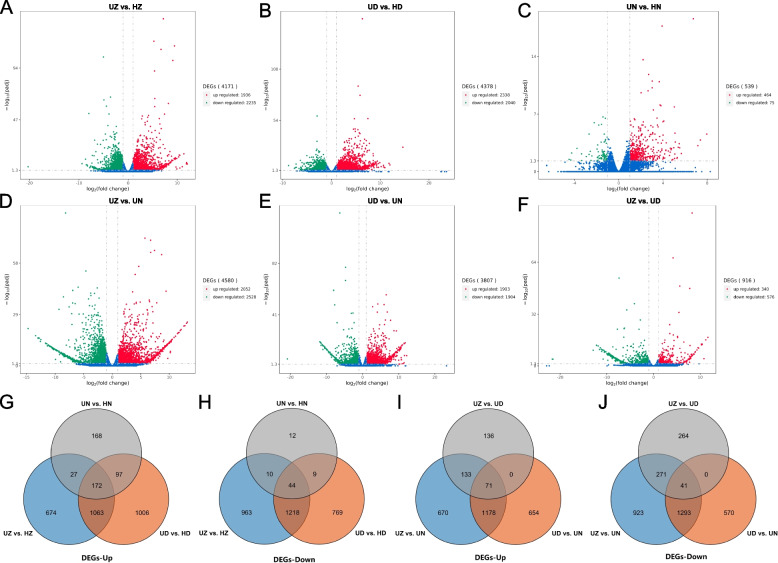
Differentially expressed genes (DEGs) in 6 groups (UZ vs. HZ, UD vs. HD, UN vs. HN, UZ vs. UN, UD vs. UN and UZ vs. UD). Red color represents up-regulated DEGs, green color represents down-regulated DEGs, and blue color represents non-differentially expressed genes. **A** Volcano plot showing DEGs in UZ vs. HZ. **B** Volcano plot showing DEGs in UD vs. HD. **C** Volcano plot showing DEGs in UN vs. HN. **D** Volcano plot showing DEGs in UZ vs. UN. **E** Volcano plot showing DEGs in UD vs. UN. **F** Volcano plot showing DEGs in UZ vs. UD. **G** Venn diagram of up-regulated DEGs in UZ vs. HZ, UD vs. HD and UN vs. HN and overlapping of the three groups. **H** Venn diagram of down-regulated DEGs in UZ vs. HZ, UD vs. HD and UN vs. HN and overlapping of the three groups. **I** Venn diagram of up-regulated DEGs in UZ vs. UN, UD vs. UN and UZ vs. UD and overlapping of the three groups. **J** Venn diagram of down-regulated DEGs in UZ vs. UN, UD vs. UN and UZ vs. UD and overlapping of the three groups

We firstly analysed differential gene expression between virus-infected barley and healthy barley (the barley grown in the disease-free field and exhibiting no symptoms of typical BaYMV disease). Differentially expressed genes (DEGs) were identified through comparing differential expression levels of genes in BaYMV and BaMMV infected Zaoshu 3 (UZ) and healthy Zaoshu 3 (HZ), thereby identifying genes associated with BaYMV and BaMMV infection (adjusted *p*-value < 0.05 & log2|Fold Change|> 1). It can be found that 1,936 genes out of the total 4,171 significant DEGs were up-regulated while 2,235 DEGs were down-regulated (Fig. 2A). Next, we compared differential expression levels of genes in BaMMV-infected UD and healthy HD. There were 4,378 genes differentially expressed with significance, of which 2,338 genes were up-regulated while 2,040 genes were down-regulated (Fig. 2B). Finally, gene expression levels of BaYMV and BaMMV-resistant samples were compared between disease soils (UN) and healthy soils (HN). The result revealed 464 genes were up-regulated and 75 genes were down-regulated (Fig. 2C).

When DEGs were compared among all the above 3 groups, 172 up-regulated DEGs and 44 down-regulated DEGs were in common. These common genes expressed in different genetic backgrounds may affect the occurrence of BaYMV disease. In contrast to the common DEGs, 674 up-regulated and 963 down-regulated DEGs were only specific in UZ but not in HZ, 1006 up-regulated and 769 down-regulated DEGs were only specific in UD but not in HD and 168 up-regulated and 12 down-regulated DEGs were only specific in UD but not in HD (Fig. 2G; H). After excluding the genetic background, 1235 and 1262 genes were differentially up-regulated and down-regulated in group (UZ vs. HZ) and another group (UD vs. HD), respectively. Among all the 3 groups, group (UD vs. HD) contains the most up-regulated DEGs (total 2,338 DEGs), while group (UN vs. HN) contains the least up-regulated DEGs (total 464 DEGs) (Fig. 2G). In terms of down-regulated DEGs, group (UZ vs. HZ) contains the most (total 2,235 down-regulated DEGs), while the group (UN vs. HN) contains the least (total 75 down-regulated DEGs) (Fig. 2H).

### Differential gene expression between the infected barley cultivars

To identify significant differentially expressed genes of 3 varieties in disease soils, we performed DEG analysis and drew volcano plots with the criteria of adjusted *p*-value < 0.05 & log2|Fold Change|> 1. Among the disease-susceptible varieties (UZ and UD) and disease-resistant variety (UN) in disease soils, we identified 2,052 up-regulated DEGs and 2,528 down-regulated DEGs between UZ and UN (Fig. 2D), 1,903 up-regulated DEGs and 1,904 down-regulated DEGs between UD and UN (Fig. 2E), and 340 up-regulated DEGs and 576 down-regulated DEGs between samples infected with BaYMV and BaMMV (UZ) and samples infected with BaMMV (UD) (Fig. 2F).

We further analyzed the types and numbers of DEGs in disease soils using the Venn diagram. The result showed that 71 up-regulated and 41 down-regulated DEGs were in common among all the 3 groups (UZ vs. UN, UD vs. UN and UZ vs. UD) (Fig. 2I; J). Besides, 670 up-regulated and 923 down-regulated DEGs were specific in UZ vs. UN, 654 up-regulated and 570 down-regulated DEGs were specific in UD vs. UN, and 136 up-regulated and 264 down-regulated DEGs were specific in UZ vs. UD (Fig. 2I; J).

### Virus infection responsive or associated genes

**Fig. 3:**
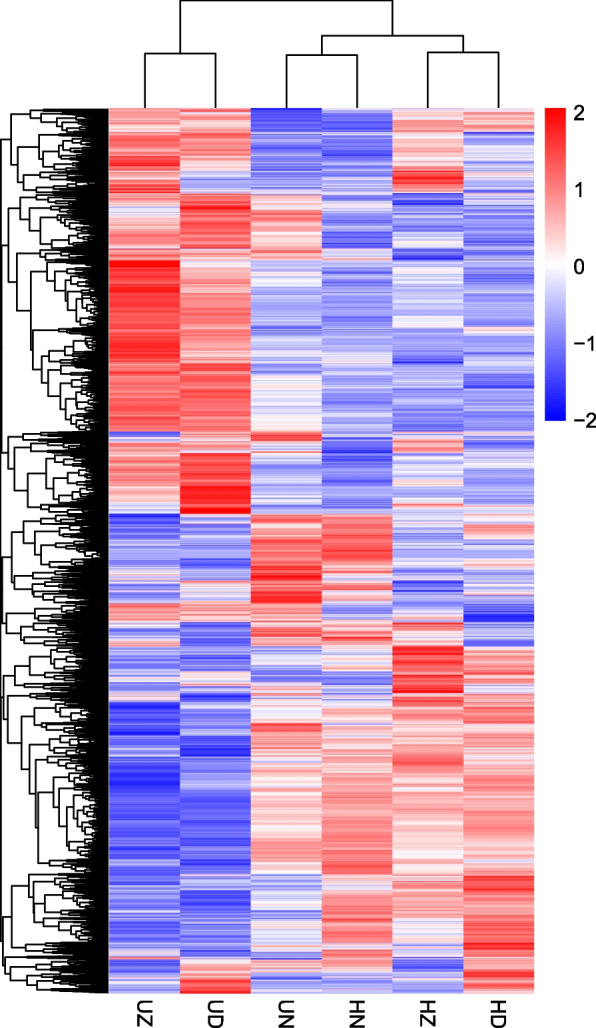
Hierarchical clustering heat map of all DEGs. The red color represents the high expression abundance and the blue color represents the low expression abundance

To illustrate virus infection responsive or associated genes, hierarchical clustering analysis was performed through calculating the FPKM values and log2 (FPKM + 1) (Fig. 3). The genes differentially expressed between the infected samples and the uninfected samples are clearly displayed in the heatmap. A total of 3 distinct clusters are viewed of all the samples (Fig. 3). One cluster includes UZ and UD, one includes UN and HN, and one includes HZ and HD. In terms of DEGs, as many as 13 main clusters were found (Fig. 3).

**Fig. 4:**
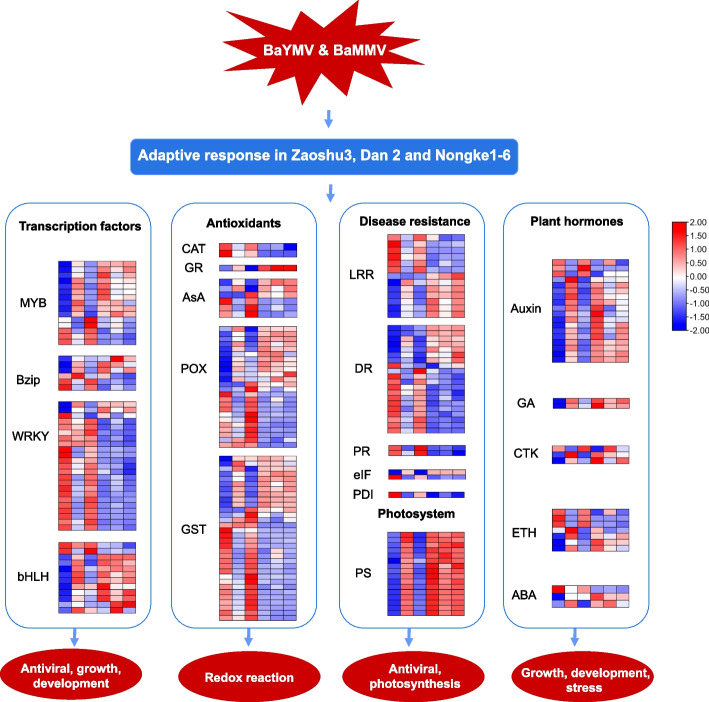
A hypothetical model of gene response to BaYMV disease. Different colors represent different genes. Relative expression levels were normalized and displayed as a color gradient from low (blue) to high (red). In each heatmap samples from left to right are UZ (first column), HZ (second column), UD (third column), HD (fourth column), UN (fifth column), and HN (sixth column)

In total, 192 genes were differentially expressed after BaYMV/BaMMV infection. Some of these genes were involved in transcriptional regulation, anti-oxidation, disease resistance, and plant hormone deserve attention (Fig. 4). A total of 56 DEGs were identified to encode different families of transcription factors (TFs), such as MYB (15), Bzip (6), WRKY (23), and bHLH (12). It is assumed that these transcription factors are to be related by the virus infection. The expression of the genes encoding antioxidants may be related to the symptoms of yellowing on barley leaves. These antioxidants-encoding genes were mainly the genes encoding superoxide dismutase (SOD), catalase (CAT), ascorbic acid specific peroxidase (APX), and glutathione reductase (GR). Altogether, 65 major antioxidants were found in this study. For disease resistance genes, 13, 20 and 2 DEGs belonging to LRR-repeat protein, disease resistance protein, and pathogenesis-related protein, respectively, were identified (Fig. 4). Two genes (HORVU5Hr1G051970, HORVU7Hr1G122100) encoding pathogenesis-related genes were the most significantly differentially expressed. It was additionally found that disease resistance genes and LRR genes varied in less expression level than pathogenesis-related genes. It is worth mentioning that genes *eIF4E* and *PDIL5-1*, which encode eukaryotic translation initiation factor and protein disulfide isomerases, respectively, that had been previously cloned were further investigated for their differential expression in susceptible samples (Fig. 4). For the genes related to plant hormones, 33 DEGs were identified, which were involved in plant hormone signal transduction pathways, including auxin (IAA), gibberellin (GA), cytokinin (CTK), abscisic acid (ABA), ethylene and polyamine (Fig. 4). In general, the susceptible variety showed greater changes than the resistance variety after virus infection.

### GO functional analysis

The identified differentially expressed genes were further analysed by Gene ontology (GO) enrichment. All the genes were classified into three categories: molecular function (MF), cellular component (CC), and biological process (BP) (Fig. S1A). In details, 9,875 target genes from UZ vs. HZ were assigned to 770 Go pathways. Small molecule metabolic process (GO:0,044,281), carbohydrate metabolic process (GO:0,005,975), response to external biotic stimulus (GO:0,043,207), defense response to other organisms (GO:0,098,542), catabolic process (GO:0,009,056) were significantly enriched. In UD vs. HD, 15,395 target genes were assigned to 770 Go pathways. Translation (GO:0,006,412), cellular amide metabolic process (GO:0,043,603), organonitrogen compound biosynthetic process (GO:1,901,566), protein-containing complex (GO:0,032,991), structural molecular activity (GO:0,005,198), structural constituent of ribosome (GO:0,003,735) were significantly enriched. In UN vs. HN, 2,160 target genes were assigned to 457 Go pathways. Organonitrogen compound biosynthetic process (GO:1,901,566), protein-containing complex (GO:0,032,991) and cytoplasmic part (GO:0,044,444) were significantly enriched. In UZ vs. UN, 7,130 target genes were assigned to 747 Go pathways. Cytoplasmic parts (GO:0,044,444), carbohydrate binding (GO:0,030,246) and ADP binding (GO:0,043,531) were enriched in large numbers. In UD vs. UN, 12,015 target genes were assigned to 774 Go pathways. Defense response (GO:0,006,952), protein folding (GO:0,006,457), response to biotic stimulus (GO:0,009,607) and photosystem (GO:0,009,521) were enriched. In UZ vs. UD, 732 target genes were assigned to 313 Go pathways. However, only ADP binding (GO:0,043,531) was significantly enriched.

To obtain more biological information about barley yellow mosaic disease, KEGG enrichment of differentially expressed genes was also conducted. Top 20 significantly enriched KEGG pathways were identified (Fig. S1B). In comparison among the six groups (UZ vs. HZ, UD vs. HD, UN vs. HN, UZ vs. UN, UD vs. UN and UZ vs. UD), 1,132, 1,780, 289, 693, 1506 and 98 target genes were assigned to 108, 112, 88, 107, 110 and 66 KEGG pathways, respectively.

### Validation of sequencing data by quantitative PCR

**Fig. 5:**
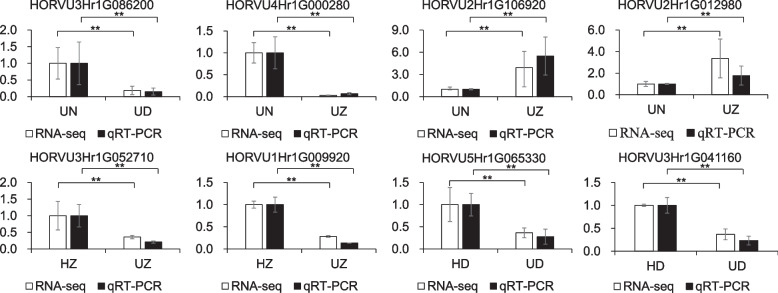
Quantitative real-time PCR validation of 8 differentially expressed genes and the corresponding expression data of RNA-Seq. The black filled columns represent relative expression levels obtained by qRT-PCR, and white filled columns represent relative expression obtained by RNA-Seq

To validate differentially expressed genes identified in the RNA sequencing data, 8 DEGs were selected for qRT-PCR analysis (Fig. 5). These DEGs were HORVU3Hr1G086200, HORVU4Hr1G000280, HORVU3Hr1G052710, HORVU1Hr1G009920, HORVU3Hr1G041160, HORVU5Hr1G065330, HORVU2Hr1G106920 and HORVU2Hr1G012980. It appeared that expression levels of HORVU3Hr1G086200, HORVU4Hr1G000280, HORVU3Hr1G052710, HORVU1Hr1G009920, HORVU3Hr1G041160 and HORVU5Hr1G065330 decreased while expression levels of HORVU2Hr1G106920 and HORVU2Hr1G012980 increased, after BaYMV or BaMMV infection. This qRT-PCR result was consistent with the transcriptome data.

## Discussion

High-throughput RNA sequencing provides a comprehensive transcriptome profile in plants, important in elucidating plant-infecting partitiviruses mechanisms [20, 21]. By comparing transcriptomes of virus-infected vs. virus-uninfected plants, genes associated with virus resistance can be identified, serving as critical genetic resources in breeding crops resist to viral diseases [22, 23]. In our study, we utilized the RNA-seq technology and thoroughly analysed transcriptome profiles of disease-resistant varieties, BaYMV & BaMMV susceptible varieties and BaMMV susceptible varieties grown under diseased and non-diseased field conditions. GO enrichment and KEGG pathway analysis displayed that the class of “peptidase complex”, “proteasome complex” and “endopeptidase complex” were highly represented in “UZ vs. HZ”, “UD vs. HD” and “UD vs. UN”, including Proteasomal degradation as a member of this class. Proteasomal degradation is known to be an important mechanism of the host controlling virus infection [24]. It is involved in the immune response, the resistance response, the induction of systemic acquired resistance and the host ubiquitination pathway to resist or limit pathogen infestation [25]. In cells, the ubiquitin–proteasome system (UPS) is responsible for protein degradation [26]. Highly expressed genes involving proteasomal degradation suggests that plant viruses could regulate the expression of UPS genes or E3-ubiquitin ligases activity, affect proteasome activity directly, and determine the ubiquitination status of host cells, as previously reported [27]. Furthermore, the UPS plays a key role involving in a series of steps from pathogen perception to downstream signalling to modulate plant defense [28]. Therefore, the genes in the class of “peptidase complex”, “proteasome complex” and “endopeptidase complex” may be associated with the pathogenic mechanism of BaYMV disease and engage in arms races between hosts and viruses. Another interesting finding in the KEGG pathway analysis is that “protein processing in endoplasmic reticulum” was significantly enriched in UZ vs. HZ and UZ vs. UN. Endoplasmic reticulum stress is the initial cellular response to stress [29]. This finding suggests that the “protein processing in endoplasmic reticulum” pathway might be activated after BaYMV and BaMMV infection.

TFs play an important role in biotic stress such as pathogen infection and insect attacks. Based on the differential expression analysis of BaYMV/BaMMV-infected vs. healthy libraries (Fig. 3), 36 DEGs were found to be similar in expression level (up- or down-regulated) in both groups. These include MYB genes, which are involved in antiviral processes in plants. Inhibition of NtMYB1 in tobacco weakens N-gene-mediated resistance to tobacco mosaic virus (TMV) [30]. Other TFs include the bZIP protein involved in the transcriptional regulation of rice tungro bacilliform virus (RTBV) promoter after virus infection in rice [31]. WRKY, a key regulatory factor in plant immune response to various biological stresses and having the most conserved amino acid domain [32], CaWRKYd regulating hypersensitive response (HR) cell death by regulating pathogenesis-related (PR) gene expression when Pepper mild mottle virus (PMMoV) infected Hot pepper [33], and SlybHLH131 associated with Tomato yellow leaf curl virus (TYLCV) infection when Tomato yellow leaf curl virus (TYLCV) infected tomatoes [34]. These findings support that TFs are involved in the response process of barley yellow mosaic disease although at different degrees. Further exploring mechanisms of TFs in the response to BaYMV disease is necessary.

Plants maintain their redox homeostasis through an efficient antioxidative system composed of both enzymatic and nonenzymatic antioxidants [35]. Among these enzymes mainly are catalase (CAT), glutathione reductase (GR), ascorbic acid (AsA), peroxidase (POX), and glutathione S-trans-ferase (GST). When plants are attacked by pathogens, they resort to oxidative bursts involving oxygen species such as CAT. For example, Chilli veinal mottle virus (ChiVMV) can interact directly with two catalases in tobacco to promote virus replication [36]. In this study, two genes (HORVU6Hr1G008640 and HORVU6Hr1G008730) were highly expressed in the two susceptible samples, supporting that catalase was involved in the initiation of the plant body defense system. However, a GR gene (HORVU4Hr1G073930) was higher in tolerant varieties (UN) than in the two susceptible samples (UZ and UN), which is opposite to CAT expression in these samples. Previous study showed that Cucumber mosaic virus (CMV) movement protein (MP) can enhance virus infection and reduce redox reaction through interaction with ascorbate peroxidase [37]. Peroxidase plays a potent role in defending against pathogens in host defense, such as involving in reactive oxygen species metabolic processes, forming lignin, decomposing auxin and oxidizing some substances. The resistance of rice to southern rice black streak dwarf virus (SRBSDV) was increased by enhancing peroxidase activity after treatment with chitosan oligosaccharide, a plant immunomodulator [38]. Peroxisome-targeted effector proteins were induced in the early stage of the *M.oryzae* infection and positively regulated pathogenicity [39]. In this study, we found significant differences in ascorbate oxidase in response to BaYMV disease between resistant and susceptible plants and that peroxidase levels in infected barley leaves were up-regulated or down-regulated to varying degrees compared with uninfected leaves. Apart from the above genes, found two GST genes, HORVU2Hr1G063460 and HORVU0Hr1G019300, were most down-regulated and up-regulated in either susceptible or resistant plants. In Arabidopsis thaliana GST genes, AtGSTU19 and AtGSTU24 had been shown to be regulators of Arabidopsis thaliana in response to Turnip mosaic virus (TuMV) [40].

Chloroplasts are the core of signal integration from PTI and ETI and are obvious targets for effector regulation [41]. Potato virus Y (PVY) infection usually alters the chloroplast number of the host plant, alters host morphology, and inhibits photosynthesis [42]. The non-structural protein P5-2 from rice black-streaked dwarf virus (RBSDV) specifically targets at chloroplasts and may control the development of viral symptoms [43]. Similar situation was observed in this study for BaYMV disease infection which also significantly reduced the expression level of photosystem-related genes, thereby suggesting an involvement in viral symptom development and metastasis of the infected barley (Fig. 5). When comparing the samples from diseased fields with those from non-diseased fields, we found that the fold changes of photosystem differential genes in the resistant variety were significantly smaller than those in the susceptible varieties, indicating that the gene downregulation in the susceptible varieties was more active than in the resistant variety. In addition, it also indirectly demonstrated that the photosynthetic mechanisms in the BaYMV/BaMMV-resistant barley plants might function more efficiently than in the susceptible barley cultivars.

The Plant R gene is the most common nucleotide-binding leucine-rich repeat gene (NBS-LRR), which plays an important role in plant disease resistance [44]. After PVX infection in potato, CC-NBS-LRR protein and its elicitor PVX coat protein (CP) were co-expressed, resulting in a rapid hypersensitive response [45]. The resistance of potato to potato virus X mediated by Rx gene (CNL) is strong. Tobacco mosaic virus resistance is mediated by N gene which causes a hypersensitivity response [46]. In the present study, we discovered disease resistance-associated DEGs, whose expression levels differed between infected and non-infected samples, suggesting that these DEGs may have the ability to adjust downstream targets in both positive and negative directions. Systemic acquired resistance leads to systemic and broad-spectrum resistance, which is characterized by increased expression of pathogenesis-related genes [47]. In our study, pathogenesis-related genes were also found to be differentially expressed, and GO and KEGG analysis also enriched the plant-pathogen interaction pathway (Fig. 5). Notably, most PR genes were highly expressed in infected samples compared with the uninfected samples. Previous study showed that the activation of PR genes responds to different stresses in different plant families. Pathogenesis-related protein 10 (PR-10) gene can be upregulated in the presence of RNA4 after beet necrotic yellow vein virus (BNYVV)-infected *N. benthamiana* and is closely associated with symptom development [48]. Although we also found the expression differences of eIF and PDI-related families, significant sequence differences of two genes from the varieties were not identified.

Virus-infected plants can cause developmental abnormalities such as stunting and leaf patches and host hormone pathways may be modulated by viral infection. Exposed to pathogen infection, hormonal signals including auxin, gibberellin, cytokinin, abscisic acid, and ethylene are critical in response to pathogens. Previous studies have reported that maize infected with sugarcane mosaic virus (SCMV) increased the expression levels of the auxin binding protein 1 (ABP1) [49], and the interaction between P7-2 encoded by rice black stripe dwarf virus (RBSDV) and the host GA-insensitive dwarf protein F2 (GID2) regulate the GA signalling pathway [50]. Other host hormone pathways modulated by viral infection include cytokinin activities and ABA regulations. In white clover mosaic potexvirus-infected kidney bean plants cytokinin activities decrease [51], while ABA is upregulated during bamboo mosaic virus (BaMV) infection of *N. benthamiana* and downregulated during rice black-streaked dwarf virus (RBSDV) infection of rice [52, 53]. In addition, the ET signaling pathway is also crucial for the pathophysiological regulation of immune responses to potato virus Y (PVY) infection [54]. Consistent with these previous studies, the encoded auxin, gibberellin, cytokinin, abscisic acid, and ethylene in infected barley varieties with BaYMV and/or BaMMV and in healthy barley varieties showed significantly different expression in level, suggesting that the phenotypic characteristics of BaYMV disease were affected by plant hormones.

## Materials and methods

### Plant samples

Virus-infected samples were obtained from a barley nursery in Yangzhou, Jiangsu Province of China, where it is continually used to screen barley cultivars for BaYMV disease resistance. In March 2021, three barley varieties, Zaoshu 3 (UZ), Dan 2 (UD) and Nongke 1–6 (UN) were collected from the diseased nursery. Meanwhile, virus-free healthy plants of the same cultivar Zaoshu 3 (HZ), Dan 2 (HD) and Nongke 1–6 (HN) were obtained from a disease-free field as a control. Both the diseased and non-diseased fields are in the experimental field of Yangzhou University, and the distance between the two fields is less than 100 m. The stem and jointing of the sample collected were developing, and the infected sample showed typical symptoms of BaYMV disease (about before jointing). Under diseased nursery, Zaoshu 3 cultivar was co-infected with BaYMV and BaMMV, Dan2 cultivar was infected with BaMMV, and Nongke 1–6 cultivar was not infected with BaYMV and BaMMV. Each sample contained three biological replicates.

### RNA extraction, library construction and sequencing

We extracted total RNA using the total RNA rapid extraction kit for polysaccharides poolyphenol plant (Tiangen, China) according to the manufacturer's instructions. Analyzing RNA integrity was performed on the Bioanalyzer 2100 system using the RNA Nano 6000 Assay Kit. One microgram RNA per sample was used for library construction as below the steps. First-strand cDNA was synthesized using a random hexamer primer and M-MuLV Reverse Transcriptase. Second-strand cDNA synthesis was subsequently performed using DNA Polymerase I and RNase H. Phusion High-Fidelity DNA polymerase, Universal PCR primers and Index (X) Primer were used for PCR. After purification, PCR products were evaluated for their qualities with Agilent bioanalyzer 2100. Sequencing of the PCR products was performed using Illumina NovaSeq 6000 platform.

### Bioinformatics analyses

Clean reads were obtained by removing reads containing adapter, low-quality reads and reads containing ploy-N from raw data. Contents of Q20, Q30, and GC contents were calculated. Next, clean reads were aligned to the barley reference genome using HISAT2 [55]. Transcript abundance in each sample was estimated by StringTie [55]. This process involved the use of a novel network flow algorithm, as well as an optional de novo assembly step, to accurately assemble and quantitate full-length transcripts that represent multiple splice variants for each gene locus. We conducted differential expression analysis between two conditions/groups using the DESeq2 R package. FeatureCounts was used to calculate the readings mapped to each gene. The FPKM (fragments per kilobase of transcript per million mapped reads) of each gene was calculated based on its length and the count mapped to that gene is read. The *P*-values were adjusted using the Benjamini & Hochberg method, and a adjusted *p*-value threshold of 0.05 and |log2fold change|> 1 were established as the criteria for significant differential expression. Gene Ontology (GO) enrichment analysis of DEGs' response to BaYMV disease was performed by clusterProfiler R package to understand DEGs' functions. We considered differentially expressed genes to be significantly enriched for GO terms or KEGG pathways with corrected *p*-values less than 0.05. Additionally, we used the clusterProfiler R package to test whether KEGG pathways were statistically enriched for differentially expressed genes [56].

### Quantitative Reverse Transcription Polymerase Chain Reaction (qPCR)

qPCR analysis of mRNA expression was performed using cDNAs synthesizedwith HiScript® III RT SuperMix (cat. no. R323; Vazyme Biotech Co., Ltd.) according to the manufacturer's instructions. ChamQ Universal SYBR® qPCR Master Mix (cat. no. Q711; Vazyme Biotech Co., Ltd) was used in qPCR with reaction conditions as follows: firstly 95 °C for 30 s, then 95 °C for 10 s for 40 cycles and finally 60 °C for 30 s. A CFX96 Touch™ Real-Time PCR Detection System (Bio-Rad, Hercules, CA, USA) was used for qRT-PCR in 96-well plates. *GADPH* and *α-Tubulin* were used as two reference genes. All the primers used in this study are listed (Table S2). The qPCR experiments encompassed three independent biological replicates. Relative gene expression levels were analysed using the comparative 2^−ΔΔCT^ method [57, 58].

## Conclusions

Barley yellow mosaic disease poses a serious threat to winter barley production. It is critical to understand the differentially expressed genes in response to barley yellow mosaic disease in both resistant and susceptible cultivars. In this research, we utilised high-throughput sequencing and thoroughly examined transcriptome changes in barley upon infection with BaYMV and/or BaMMV. Gene Ontology and KEGG analysis indicated significant enrichments in the peptidase complex and protein processing in endoplasmic reticulum. Differentially expressed genes found between infected and uninfected barley varieties are clustered into protein families encoding transcription factors, antioxidants, disease resistance genes, and hormones. The main resistance sources of BaYMV disease have failed, so it is necessary to explore and utilize new resistance sources for barley disease breeding. This study laid a foundation for the exploration of new genes resistant to BaYMV disease. It will contribute to the development of barley varieties with high resistance to disease, rescuing the economic loss caused by BaYMV disease.

## Supplementary Information

**Additional file 1: Figure S1.** Function analysis of all DEGs in 6 groups. A: Gene ontology function enrichment response to BaYMV disease. B: KEGG pathway enrichment response to BaYMV disease.

**Additional file 2: Table S1.** Alignment of the region with the barley genome.

**Additional file 3: Table S2.** Primers used in this study.

## Data Availability

The mRNA sequencing data for both infected and non-infected barley samples were deposited in the Gene Expression Omnibus (GEO) database at the NCBI under accession number GSE225976.
