# Supplementary material for: Molecular insights into the responses of barley to yellow mosaic disease through transcriptome analysis
Source: BMC Plant Biol. 2023 May 19;23:267. doi: 10.1186/s12870-023-04276-x (PMC10197257; doi:10.1186/s12870-023-04276-x)
Supplement: Supplementary file 1 — Additional file 1: Figure S1. Function analysis of all DEGs in 6 groups. A: Gene ontology function enrichment response to BaYMV disease. B: KEGG pathway enrichment response to BaYMV disease. [file 12870_2023_4276_MOESM1_ESM.pdf]

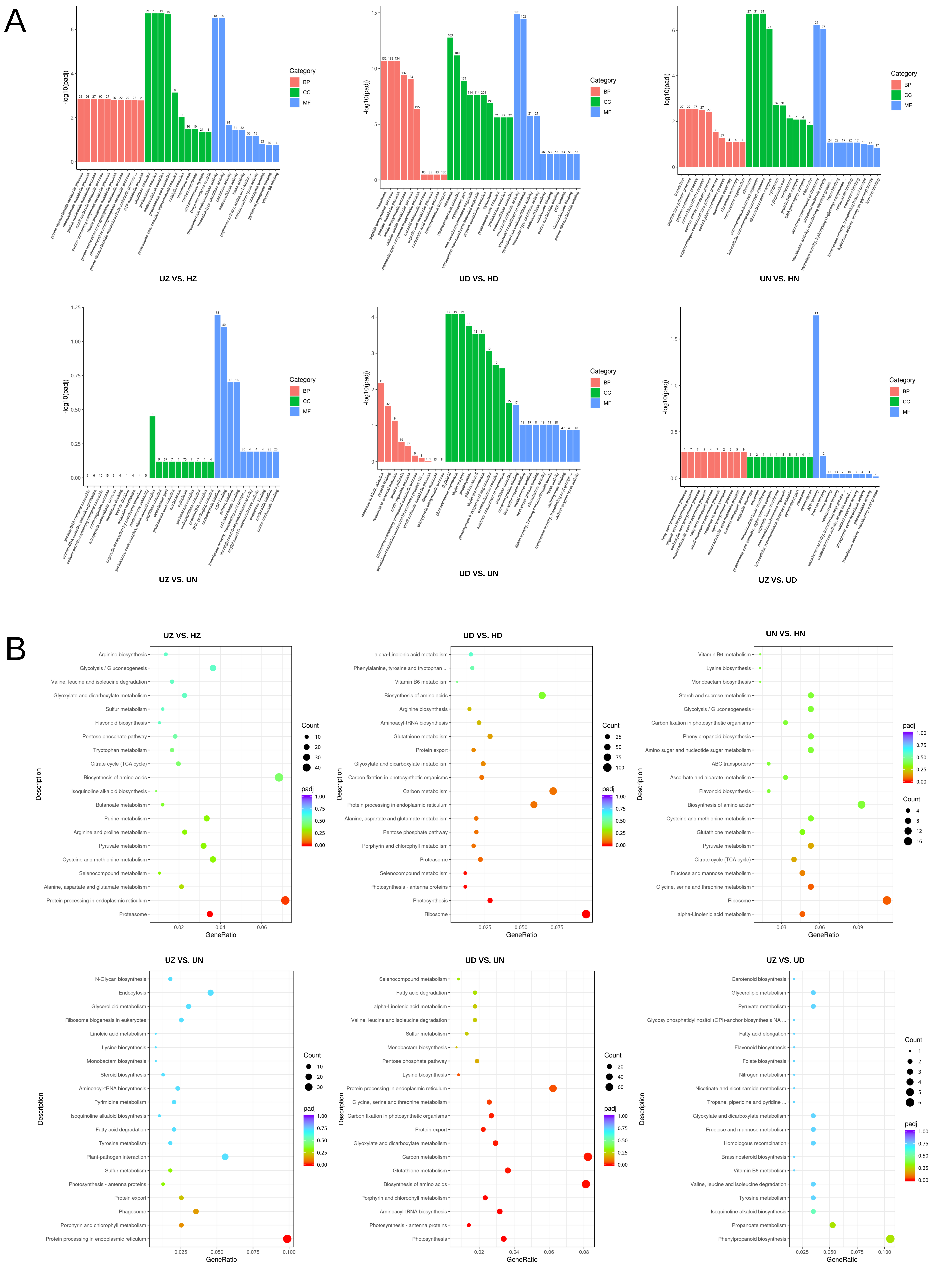

Figure S1. Function analysis of all DEGs in 6 groups.
A: Gene ontology function enrichment response to BaYMV disease.
B: KEGG pathway enrichment response to BaYMV disease.
