# Supplementary material for: Molecular insights into the responses of barley to yellow mosaic disease through transcriptome analysis
Source: BMC Plant Biol. 2023 May 19;23:267. doi: 10.1186/s12870-023-04276-x (PMC10197257; doi:10.1186/s12870-023-04276-x)
Supplement: Supplementary file 2 — Additional file 2: Table S1. Alignment of the region with the barley genome. [file 12870_2023_4276_MOESM2_ESM.pdf]

Table S1. Alignment of the region with the barley genome.

sample	exon	exon_per	intron	intron_per	intergenic	intergenic_per
UZ1	5,770,606,120	91.49%	93,393,554	1.48%	443,330,574	7.03%
UZ2	5,628,199,013	90.95%	102,684,123	1.66%	457,622,616	7.39%
UZ3	4,881,419,909	90.61%	97,188,216	1.80%	408,521,139	7.58%
HZ1	5,858,259,829	90.89%	106,548,118	1.65%	480,389,022	7.45%
HZ2	5,946,566,489	90.98%	109,742,539	1.68%	479,549,949	7.34%
HZ3	5,863,284,357	90.63%	112,057,053	1.73%	494,106,180	7.64%
UD1	5,738,732,645	90.85%	103,074,789	1.63%	474,660,639	7.51%
UD2	5,602,231,091	91.18%	106,774,455	1.74%	435,022,592	7.08%
UD3	6,603,566,694	90.57%	127,795,901	1.75%	559,800,697	7.68%
HD1	6,170,259,077	91.66%	104,076,463	1.55%	457,168,359	6.79%
HD2	6,542,870,556	90.00%	138,626,594	1.91%	588,344,934	8.09%
HD3	6,652,227,773	90.40%	148,629,624	2.02%	557,927,410	7.58%
UN1	5,936,810,214	91.38%	99,847,512	1.54%	459,835,294	7.08%
UN2	6,126,245,031	91.45%	102,371,729	1.53%	470,697,932	7.03%
UN3	5,993,608,855	91.11%	108,127,521	1.64%	476,675,690	7.25%
HN1	5,836,024,708	91.03%	108,468,075	1.69%	466,585,067	7.28%
HN2	5,772,324,678	89.59%	146,005,780	2.27%	524,479,528	8.14%
HN3	6,048,101,142	90.92%	123,930,074	1.86%	480,254,020	7.22%
