# Supplementary material for: Molecular insights into the responses of barley to yellow mosaic disease through transcriptome analysis
Source: BMC Plant Biol. 2023 May 19;23:267. doi: 10.1186/s12870-023-04276-x (PMC10197257; doi:10.1186/s12870-023-04276-x)
Supplement: Supplementary file 3 — Additional file 3: Table S2. Primers used in this study. [file 12870_2023_4276_MOESM3_ESM.pdf]

Table S2. Primers used in this study.

Gene	Primer
GADPH-F	GTGAGGCTGGTGCTGATTACG
GADPH-R	TGGTGCAGCTAGCATTTGAGAC
α-Tubulin-F	AGTGTCCTGTCCACCCACTC
α-Tubulin-R	AGCATGAAGTGGATCCTTGG
qBaYMV-F	AACAATGGCACCTCAGACAAGCAT
qBaYMV-R	CCTTCGGAGTCCACCATTCATTCG
qBaMMV-F	TGGCATCACCGACGACGAGAA
qBaMMV-R	CTGAGACCGCCATTGACTTCCATAG
HORVU3Hr1G086200-F	TCATCTTCGGTGAATGGTGGTTGTC
HORVU3Hr1G086200-R	TGTCTTTGGCGGAGCAGTCGTA
HORVU4Hr1G000280-F	GAGGAGACGGAGTGCTGCTCTT
HORVU4Hr1G000280-R	CCTTGTCGCCTTCCAATGAATCAGA
HORVU2Hr1G106920-F	ACCGACAGTGTCATCGTCAACCT
HORVU2Hr1G106920-R	TGCCTCCTCCTTCTCCACCTCT
HORVU2Hr1G012980-F	CGAGAAGACGACGACACGGTAAC
HORVU2Hr1G012980-R	GCACGGACCAAGCAAGTGATGT
HORVU3Hr1G052710-F	TTCTGAGCCGCTGACCAACA
HORVU3Hr1G052710-R	GATGCACCCGACGCCGACAA
HORVU1Hr1G009920-F	CACCGACATCATCCATGACCCC
HORVU1Hr1G009920-R	GCGGAGCACCTGCACCACCA
HORVU5Hr1G065330-F	GGGTACATCGCGTCGTGGCT
HORVU5Hr1G065330-R	GTGCATTCTTCGGGTCATCTGG
HORVU3Hr1G041160-F	CTTGAGGATAATATCTTCCGAATC
HORVU3Hr1G041160-R	GGCCCTTGTTAGCATCTTTG
